# Basis for the Age-related Decline in Intestinal Mucosal Immunity

**DOI:** 10.1080/10446670310001642168

**Published:** 2003

**Authors:** Douglas L. Schmucker, Robert L. Owen, Robert Outenreath, Karine Thoreux

**Affiliations:** 1 aCell Biology & Aging Section Veterans Affairs Medical Center University of California San Francisco CA USA; 2 Department of Anatomy University of California San Francisco CA USA; 3 Department of Medicine University of California San Francisco CA USA; 4 Department of Epidemiology & Biostatistics University of California San Francisco CA USA; 5 Department of Psychiatry University of California San Francisco CA USA

## Abstract

The elderly are characterized by mucosal immunosenescence and high rates of morbidity and mortality associated with infectious diseases of the intestinal tract. Little is known about how the differentiation of immunoglobulin A (IgA) plasma cells in Peyer's patches (PPs) and their subsequent homing to the small intestinal lamina propria (LP) is affected by aging. Quantitative immunohistochemical analyses demonstrated a 2-fold increase in the number of IgA^+^ cells in the PPs, coupled with significant declines in the numbers of IgA^+^ and antibody-positive cells in the intestinal LP of senescent rats compared to young adult animals. These data suggest that aging diminishes the emigration of IgA immunoblasts from these lymphoid aggregates, as well as their migration to the intestinal LP. Flow cytometry and lymphocyte adoptive transfer studies showed 3- to 4-fold age-related declines in the homing of antibody-containing cells and mesenteric lymph node lymphocytes to the small intestines of rhesus macaques and rats, respectively. The number of peripheral blood IgA immunoblasts expressing the homing molecule α4β7 declined 30% in senescent rats. This was accompanied by a >17% decrease in the areal density of LP blood vessels staining positive for the cell adhesion molecule MAdCAM-1. Cumulatively, declines in expression of these homing molecules constitute a substantial age-related diminution of IgA immunoblast homing potential. *In vitro* antibody secretion by LP plasma cells, i.e. antibody secreted per antibody-positive cell, remains unchanged as a function of donor age. Intestinal mucosal immunosenescence is a consequence of reduced homing of IgA plasma cells to the intestinal LP as a result of declines in homing molecule expression.


*Clinical & Developmental Immunology*, June–December 2003 Vol. 10 (2–4), pp. 167–172

Taylor & Francis healthsciences

### Basis for the Age-related Decline in Intestinal Mucosal Immunity

DOUGLAS L. SCHMUCKER[a,b,*], ROBERT L. OWEN[a,c,d], ROBERT OUTENREATH[a,e] and KARINE THOREUX[a,b,†]

[a]*Cell Biology & Aging Section, Veterans Affairs Medical Center, University of California, San Francisco, CA, USA;* [b]*Department of Anatomy, University of California, San Francisco, CA, USA;* [c]*Department of Medicine, University of California, San Francisco, CA, USA;* [d]*Department of Epidemiology & Biostatistics, University of California, San Francisco, CA, USA;* [e]*Department of Psychiatry, University of California, San Francisco, CA, USA*

The elderly are characterized by mucosal immunosenescence and high rates of morbidity and mortality associated with infectious diseases of the intestinal tract. Little is known about how the differentiation of immunoglobulin A (IgA) plasma cells in Peyer's patches (PPs) and their subsequent homing to the small intestinal lamina propria (LP) is affected by aging. Quantitative immunohistochemical analyses demonstrated a 2-fold increase in the number of $IgA^+$ cells in the PPs, coupled with significant declines in the numbers of $IgA^+$ and antibody-positive cells in the intestinal LP of senescent rats compared to young adult animals. These data suggest that aging diminishes the emigration of IgA immunoblasts from these lymphoid aggregates, as well as their migration to the intestinal LP. Flow cytometry and lymphocyte adoptive transfer studies showed 3- to 4-fold age-related declines in the homing of antibody-containing cells and mesenteric lymph node lymphocytes to the small intestines of rhesus macaques and rats, respectively. The number of peripheral blood IgA immunoblasts expressing the homing molecule $\alpha 4\beta 7$ declined 30% in senescent rats. This was accompanied by a $>17\%$ decrease in the areal density of LP blood vessels staining positive for the cell adhesion molecule MAdCAM-1. Cumulatively, declines in expression of these homing molecules constitute a substantial age-related diminution of IgA immunoblast homing potential. *In vitro* antibody secretion by LP plasma cells, i.e. antibody secreted per antibody-positive cell, remains unchanged as a function of donor age. Intestinal mucosal immunosenescence is a consequence of reduced homing of IgA plasma cells to the intestinal LP as a result of declines in homing molecule expression.

*Keywords*: IgA; Aging; Mucosal immunity; Intestinal immunity; Lymphocyte homing

### INTRODUCTION

### Intestinal Mucosal Immune System

Mucosal surfaces constitute a discrete compartment of the immune system by virtue of a different immunoglobulin isotype (immunoglobulin A, IgA), a unique process for generating an immune response and an independent lymphocyte subpopulation. The human intestine is the largest, single immune organ; it consists of $>400\,m^2$ of mucosal surface available for antigen uptake; it contains $>70\%$ of an organism's plasma cells and it produces more IgA than the organism's production of immunoglobulin G (IgG). Mucosal surfaces are the first line of immune defense and IgA antibodies neutralize toxins, block the adherence of bacteria to the epithelium and reduce the penetration of antigens across the mucosa. Mucosal immunity depends on the cooperation of lymphoid and epithelial cells to initiate and to maintain an immune response. An effective response in the intestine involves: (1) binding, uptake, transport of antigen at the mucosal surface, antigen presentation by dendritic cells or macrophages within the Peyer's patches (PPs) and IgA isotype switching, (2) maturation and homing of antigen-stimulated PP IgA B immunoblasts to the intestinal lamina propria (LP), (3) local antibody production by mature IgA plasma cells in the LP and (4) transport of IgA antibodies across the intestinal epithelium to the mucosal surface by pIg receptor. Diminished intestinal IgA antibody titers in the elderly may reflect age-associated deficits in one or more of these critical events.

### Aging and Intestinal Immunity

The elderly, i.e. $>65$ years, constitute the most rapidly growing subpopulation in the United States. The number of elderly in the US is estimated at 33 million and this number will double by 2030. This age group presents

*Corresponding author. Address: 151E, VA Medical Center, 4150 Clement Street, San Francisco, CA 94121, USA. Tel.: +1-415-221-4810. Ext. 3450. Fax: +1-415-750-6927. E-mail: coach@itsa.ucsf.edu

†Present Address: Danone Vitapole, Nautrivaleur, 15 avenue Galilee, 92350 le Plessis Robinson, France.

ISSN 1740-2522 print/ISSN 1740-2530 online © 2003 Taylor & Francis Ltd
DOI: 10.1080/10446670310001642168

the single, most significant fiscal burden to the healthcare system and has a high morbidity and incidence of infectious diseases (Wick *et al.*, 2000; Yoshikawa, 2000). In the elderly, the intestinal tract is particularly sensitive to infectious diseases, suggesting that mucosal immune defenses are compromised (Fagiolo *et al.*, 1993; Gransden *et al.*, 1994; Owen and Lew, 1995; Jeandel *et al.*, 1996; Ginaldi *et al.*, 2001; see Powers, 1992 for a review). The efficacy of many oral vaccines diminishes with increasing recipient age; further evidence of a decline in mucosal immune responsiveness (Fujihashi *et al.*, 2000). Furthermore, clinical studies have demonstrated muted intestinal IgA antibody responses to specific antigens in geriatric subjects (Beharka *et al.*, 2001; see Schmucker *et al.*, 1996 and Schmucker and Owen, 1997 for reviews).

Although the serum IgA levels are elevated in the elderly, these titers reflect the level of monomeric, non-J-chain-containing IgA that does not bind to the polymeric immunoglobulin receptor (pIgR) and is not transported to the mucosal surface as secretory IgA (Ebersole *et al.*, 1985; Penn *et al.*, 1991). There are no age-related differences in the nonspecific immunoglobulin titers in the intestinal lumen, either *in vivo* or in the medium of cultured duodenal biopsies (Arranz and Ferguson, 1992; Ginaldi *et al.*, 2001). However, nonspecific immunoglobulin levels in intestinal secretions are poor indices of the vigor of a mucosal immune response; whereas specific antibody titers are a more critical measure of mucosal immunity (Schmucker *et al.*, 1988; Taylor *et al.*, 1992; Vajdy and Lycke, 1992). For example, biliary and intestinal anti-cholera toxin (CTx) IgA antibody titers decline with increasing age in rodents and monkeys, respectively (Schmucker *et al.*, 1988; Taylor *et al.*, 1992). Clinical studies have also documented age-related declines in other mucosal IgA antibody responses (Waldmann *et al.*, 1987; Beyer *et al.*, 1989; Fujihashi *et al.*, 2000).

The following is an analysis of the effects of aging on the major events that contribute to an intestinal mucosal immune response. Most of the data presented have been generated in our laboratory; some have been published previously and are so indicated, whereas others have not been published.

### Step 1: Antigen Uptake and Processing, Isotype Switching and Emigration of IgA Immunoblasts from the PPs

There have been no substantial studies on the effects of aging on the binding or uptake of antigens by M cells or follicle-associated epithelium. Follicle weight and the yield of PP lymphocytes have been reported to decline with increasing age in mice (Kawanishi and Kiely, 1989). However, our own studies have shown that neither the number of PPs in young adult, mature and senescent rats, i.e. 15.5, 15.7 and 14.4 patches per intestine, respectively, nor the yields of lymphocytes from these lymphoid aggregates, varies with age in rats (Schmucker *et al.*, 1988). Furthermore, we are unaware of any definitive data concerning the effects of aging on the passage of antigens across the intestinal absorptive epithelium, i.e. the enterocytes.

Mucosal antigens are processed by dendritic cells or macrophages located in the PPs. The antigen-presenting capacity of dendritic cells from the skin appears to be unchanged during aging, but dendritic cells from the PPs have not been studied (Castle *et al.*, 1999; Lung *et al.*, 2000).

We are unaware of any studies that have specifically examined the efficacy of isotype switching in putative PP IgA immunoblasts as a function of age. Lycke *et al.* showed that CD19 cells are involved in the regulation of IgA isotype switching following immunization with a T-lymphocyte-dependent antigen, but there are no data concerning the effect of aging on either the number or function of this cell subpopulation (Gardby and Lycke, 2000). Shifts in intestinal B and/or T lymphocyte subpopulations or in the relative distributions of lymphocyte subsets may contribute to diminished intestinal immunity in old animals. Kawanishi and Kiely reported a loss of CD8 ($T_{suppressor}$ lymphocytes) in the PPs of old mice, whereas our flow cytometric studies did not demonstrate a shift in the size of this subpopulation in rat PPs (Kawanishi and Kiely, 1987; Daniels *et al.*, 1993a). However, our subsequent immunohistochemical analyses revealed marked age-related differences in the distribution of CD8 cells in the PPs of these old animals (Daniels *et al.*, 1993a). We also observed 2- to 3-fold age-related increases in the number of $IgA^+$ cells in the PPs of naïve and CTx-immunized rats. More recently, using ELISPOT analysis we demonstrated that the PPs of old rats contain 2-fold fewer anti-CTx-IgA secreting cells in comparison to the PPs in young adult animals (Thoreux *et al.*, 2000). Despite the marked increase in the total number of $IgA^+$ cells, these data suggest that fewer IgA immunoblasts are stimulated to produce anti-CTx antibodies in the old animals. The absence of any obvious impairment in lymphocyte switching to the IgA isotype in senescent rats, the marked increase in the population of PP $IgA^+$ cells and the infiltration of CD8 T cells into the PP follicles, suggest that T cell suppression of IgA immunoblast maturation and emigration from the PPs may contribute to mucosal immunosenescence.

### Step 2: IgA Immunoblast Homing to the Intestinal LP

We have hypothesized that the homing of IgA immunoblasts from the PPs to the intestinal LP is diminished during aging. Several lines of evidence substantiate this hypothesis. First, the number of $IgA^+$ cells in the PPs increases 2.5-fold in senescent versus young adult rats, suggesting that they fail to emigrate from these lymphoid aggregates (Daniels *et al.*, 1993a). Second, our quantitative immunohistochemical analyses demonstrated concomitant age-related declines in the numbers of $IgA^+$ ($>60\%$) and $CTx^+$ ($>50\%$) cells in rat intestinal LP

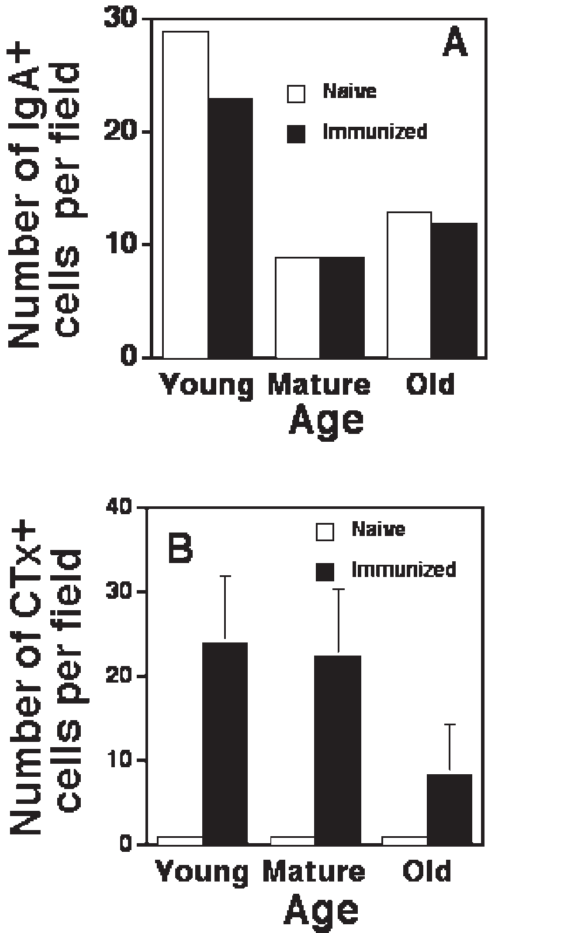

FIGURE 1 Number of (A) $IgA^+$ and (B) cholera $toxoid^+$ cells in the small intestinal LP of young (3–6 months), mature (12–14 months) and old (>24 months) male Fischer 344 rats immunized and boosted intraduodenally with cholera holotoxin (immunized) or vehicle alone (naïve). The number of $IgA^+$ cells drops markedly between young and mature animals, whereas the decline in specific antibody-containing cells occurs between maturity and senescence. Quantitative immunohistochemical analysis; each value represents the mean ± SEM for 5 animals.

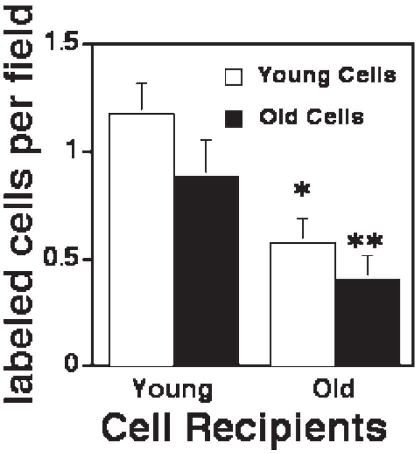

FIGURE 2 The number of PKH26 fluorescent-labeled mesenteric lymph node cells isolated from young or old donor rats, transferred intravenously into young or old recipient animals and localized to the small intestinal LP by quantitative fluorescence microscopy. The values represent the mean ± SD of fluorescent-labeled cells 20 h following adoptive transfer. The value for young cells into young recipients is significantly greater than those for either (A) young cells into old recipients (*) or (B) old cells into old recipients (**). Values represent the mean ± SEM for 4–8 animals.

following intraduodenal immunization (Schmucker *et al.*, 1988; Fig. 1). Taken together, the data from our two separate studies suggest that the emigration of putative IgA plasma cells from the PPs and their subsequent homing to the intestinal LP are compromised in senescent rats. Third, the size of these same cell populations are 3- to 4-fold lower in the peripheral blood of old rhesus macaques in comparison to young adult animals, suggesting that the number of migrating cells is diminished with age (Taylor *et al.*, 1992). Fourth, there is a marked shift of CD8 cells ($T_{suppressor}$ lymphocytes) from the interfollicular zones of PPs into B cell follicles in senescent rats, suggesting the possibility of T lymphocyte downregulation of IgA immunoblast maturation and emigration (Daniels *et al.*, 1993a). Fifth, the homing of mesenteric lymph node lymphocytes to the intestinal LP following adoptive transfer is diminished in senescent rats in comparison to young adult animals (Thoreux *et al.*, 2000; Fig. 2). The migration of fluorescent-labeled lymphocytes was lowest when old recipients received cells from old donors and greatest when young adult cells were transferred into young adult recipients. It is striking that lymphocytes from young animals exhibit reduced homing in old recipients. This observation has been confirmed by Ogino and co-workers (2001). Cumulatively, these data suggest that aging compromises the intestinal mucosal immune response at the inductive site by inhibiting the migration of IgA immunoblasts to the effector site, the intestinal LP.

Farstad *et al.* showed that IgA immunoblasts destined to leave the PPs for the intestinal LP exhibit an adhesion molecule profile of $\alpha 4b7^{high}$/L-selectin$^{low}$ (Farstad *et al.*, 1995; 1997). In an effort to identify the cause of diminished IgA immunoblast migration, we measured the expression of the gut lymphocyte homing integrin, $\alpha 4\beta 7$, on peripheral blood mononuclear cells using flow cytometry. Our study demonstrated a 30% decline in the percentage of cells expressing this critical integrin in senescent rats in comparison to young adult animals (Fig. 3). The expression of L-selectin, an integrin implicated in the homing of lymphocytes to peripheral lymph nodes and in the early steps of lymphocyte rolling and attachment to the endothelial cell adhesion molecule, MAdCAM-1, in the intestinal LP also diminishes during aging (Ogino *et al.*, 2001). Data from our quantitative immunohistochemical and stereological analysis of CD31 (all endothelial cells) and MAdCAM-1 (high endothelial venules) expression in the intestinal LP during aging in rats support our hypothesis, but are inconclusive. The intestinal LP/submucosa in old rats contained 9% more profiles of $CD31^+$ blood vessels per unit area in comparison to tissue in young adult animals; whereas the density of MAdCAM-$1^+$ vessels was 17% lower and approached statistical significance in the old rats.

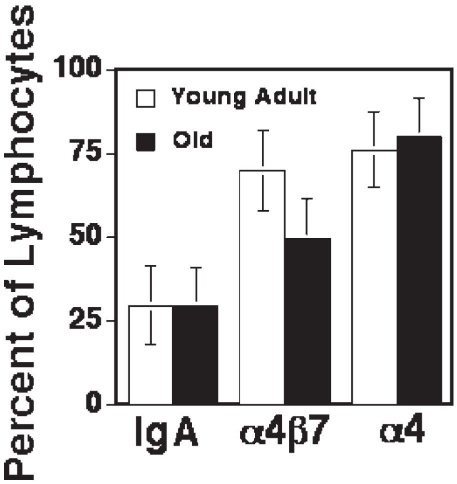

FIGURE 3 Flow cytometric analysis of the expression of the homing integrin α4β7, the integrin monomer α4 and IgA on the surface of peripheral blood mononucelar cells isolated from young and old rats. The relative percentages of cells expressing either IgA or α4 remain unchanged as a function of donor age. However, the expression of the homing molecule α4β7 declines by approximately 30% in cells from old rats (*) in comparison to those isolated from young animals ($P < 0.01$). Each bar represents the mean ± SD for 12 young or 7–8 old rats.

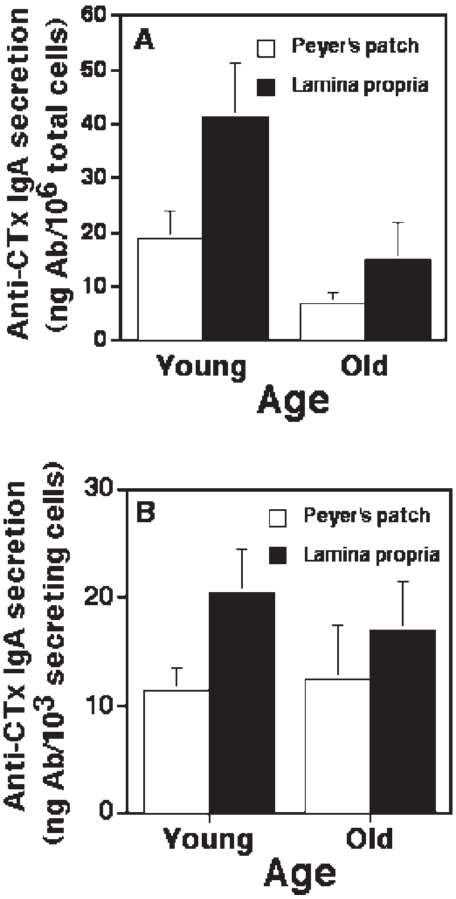

FIGURE 4 Anti-CTx IgA levels in cultures of lymphocytes isolated from PPs and small intestinal LP of young and old rats 7 days after intraduodenal boost with cholera holotoxin. The data are expressed as (A) nanograms of anti-CTx IgA antibody per $10^6$ total lymphocytes and (B) nanograms of anti-CTx IgA antibody per $10^3$ anti-CTx antibody-secreting cells. The lower antibody levels ($P < 0.05$) measured in (A) reflect fewer antibody-secreting cells per $10^6$ total lymphocytes in old rats since the antibody-secreting capacity of the specific plasma cells is undiminished with age (B). The values represent the mean ± SEM for 5 animals per age group.

These contrasting age-related shifts in the areal densities of CD1 and MAdCAM-1 stained vessel profiles yielded a 23% increase in the CD31/MAdCAM-1 areal density ratio in the old animals. The age-related declines in the expression of α4β7 and MAdCAM-1 on peripheral blood cells and intestinal LP venules, respectively, suggests a substantial diminution of the IgA immunoblast homing potential. The basis for diminished homing molecule expression in old animals is unknown, but may reflect shifts in the sensitivity to or in the levels of inflammatory cytokines, chemokines or neuropeptides (Takeuchi and Baichwal, 1995).

### Step 3: Local IgA Antibody Production

Aging is reported to result in 40–70% declines in antibody secretion by PP and intestinal LP lymphocytes isolated from mice and rats (see Schmucker *et al.*, 2001 for a review). Daniels *et al.* measured antibody secretion by gut-associated lymphoid tissue lymphocytes isolated from young and old rats following intraduodenal immunization with CTx (Daniels *et al.*, 1993b). Anti-CTx IgA antibody secretion by mesenteric lymph node lymphocytes from young animals was significantly greater than that by similar cells isolated from old rats. However, these data were based on the total number of cells in the culture and did not account for possible age-related differences in the relative number of antibody-secreting cells.

In a recent study, we demonstrated that the *in vitro* secretion of anti-CTx-IgA antibodies by lymphocytes (per $10^6$ total cells) isolated from the intestinal LP of senescent rats is significantly lower (>60%) than that of similar cells obtained from young animals (Thoreux *et al.*, 2000; Fig. 4). However, expressing antibody secretion per number of anti-CTx-IgA secreting cells (per $10^6$ specific antibody-secreting cells) eliminated this apparent age-related difference. Despite our previous finding that the number of CD8 lymphocytes in the intestinal LP increases ~2.5-fold in rats between 3 and 29 months of age, our recent data suggest that IgA antibody secretion by LP plasma cells is not suppressed in old animals. Moreover, the age-related decline in antibody titers in the intestinal lumen reflects fewer secreting cells, rather than diminished antibody secretion per cell.

Cholecystokinin (CCK), the most prevalent neuropeptide, has been shown to enhance IgA secretion into the intestinal lumen, a response that is muted by blocking the CCK receptor (Freier *et al.*, 1987; Medina *et al.*, 1998, 1999). Furthermore, CCK secretion is diminished in old rats, suggesting that a decline in this neurotransmitter/gut hormone contributes to mucosal immunosenescence (Alverdy *et al.*, 1997). On the one hand, we recently showed that $10^{-8}$ M CCK enhances *in vitro* anti-CTx IgA secretion by LP lymphocytes isolated from young adult and senescent rats by ~140% in comparison to their respective non-stimulated age cohorts. On the other hand, preliminary studies also suggest that Substance P, an intestinal neuropeptide with immunoregulatory properties, does not enhance *in vitro* anti-CTx IgA antibody secretion by LP plasma cells isolated from either young adult or senescent rats. The importance of age-related changes in the levels of immunoregulatory molecules or in the sensitivity of target lymphocytes to these molecules remains unresolved.

### Step 4: Antibody Transport Across the Intestinal Epithelium and Secretion onto the Mucosal Surface

The intestinal immune response culminates in the transport of antibodies across the intestinal epithelial cells and their secretion onto the mucosal surface, a process that requires receptor-mediated endocytosis at the basal plasma membrane and vesicle translocation to the cell's apical surface. We previously reported a 4- to 6-fold decline in the transport of polymeric IgA (pIgA) from blood to bile in rats between 3 and 25 months of age (Schmucker *et al.*, 1985). Furthermore, we showed a concomitant 3- to 4-fold decline in the number of pIgA receptors (pIgR) across this same age span (Daniels *et al.*, 1985). We and others showed that (a) the rat hepatic pIgR mRNA steady state level declines 20% between these ages and (b) the incorporation of [$^{35}$S]-cysteine into pIgR lags in cultured hepatocytes isolated from old rats in comparison to cells from young animals (Gregoire *et al.*, 1992; van Bezooijen *et al.*, 1994). Therefore, the decline in hepatic pIgR expression contributes to reduced IgA secretion into the bile and, ultimately, to a diminished intestinal immune response in rats.

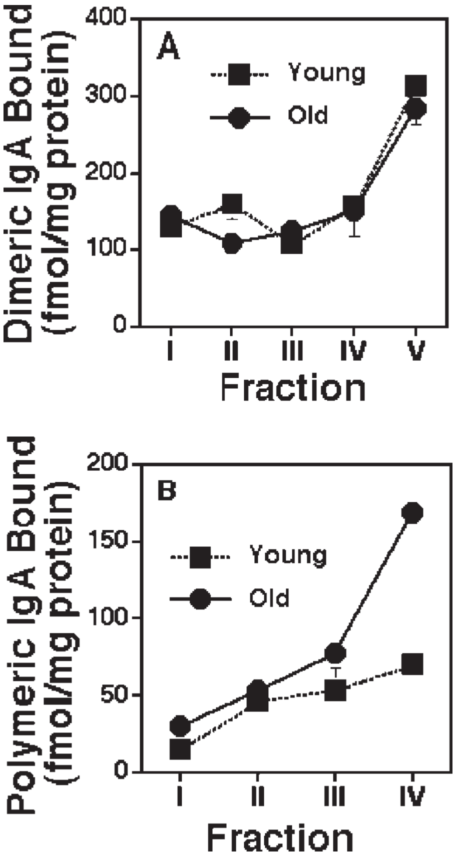

FIGURE 5 pIgR-specific binding of (A) [$^{125}$I] rat dimeric IgA to rat intestinal enterocyte basolateral plasma membranes and (B) [$^{125}$I] human polymeric IgA to rhesus macaque intestinal enterocyte basolateral membranes as a function of animal age. The increasing gradient of pIgR expression from the villus tip (Fraction I) to the crypt (Fractions IV or V) remains unchanged during aging in both models and reflects the age of the intestinal epithelial cells rather than that of the donor animals. The data reflect the mean ± SEM for 4–7 animals in each age group.

However, the hepatobiliary transport of pIgA is an important secretory pathway in only a few animals (e.g. mice, rats, rabbits). In most species, including primates, IgA antibodies are transported to the intestinal lumen across the mucosal epithelial cells by the same pIgR mechanism. Our previous study demonstrated that pIgR expression on the basolateral plasma membranes of rat small intestinal epithelial cells remains unchanged during aging (Daniels and Schmucker, 1987; Fig. 5). Crypt enterocytes exhibit a 3-fold greater binding of pIgA than do villus tip cells, but this crypt-to-villus tip gradient in pIgR expression is identical in young and old rats and a similar pattern is seen in rhesus macaques (Daniels *et al.*, 1988; Taylor *et al.*, 1992). The absence of an age-related shift in the expression of pIgR suggests that antibody binding by intestinal epithelial cells remains unscathed by aging. However, little is known about the effect of aging on steps subsequent to antibody binding by intestinal epithelial cells, i.e. endocytosis and vesicular translocation to the mucosal surface.

### CONCLUSIONS

Aging is associated with a decline in the intestinal mucosal immune response that results in diminished IgA antibody titers in the intestinal lumen and, as a consequence, marked increases in the morbidity and mortality due to infectious diseases. Intestinal immunosenescence does not result from declines in IgA antibody secretion by plasma cells in the intestinal LP or in the subsequent receptor-mediated binding of IgA to intestinal epithelial cells. Current evidence suggests that the emigration of IgA immunoblasts from the PPs and their subsequent homing to the intestinal LP is compromised in senescent rats in comparison to young adult animals. Although we have not yet established a direct causal relationship between diminished lymphocyte homing and the muted intestinal mucosal immune response in old animals, the age-related declines in the expression of critical homing molecules, especially the integrin α4β7, substantiate our hypothesis.

### *Acknowledgements*

Much of the research reported herein has been supported by the Department of Veterans Affairs, the National Institute on Aging (NIH), The American Federation for Aging Research, and the Danone Company (France). We are particularly indebted to Rose K. Wang for superb technical assistance throughout all of the studies described.

### References

Alverdy, J., Stern, E., Poticha, S., Baunoch, D. and Adrian, T. (1997) "Cholecystokinin modulates mucosal immunoglobulin A function", *Surgery* **122**, 386–393.

Arranz, E. and Ferguson, A. (1992) "Aging and gastrointestinal immunity", *Eur. J. Gastroenterol. Hepatol.* **4**, 1–4.

Beharka, A., Paiva, S., Leka, L., Ribaya-Mercado, J.D., Russell, R.M. and Meydani, S.N. (2001) "Effect of age on the gastrointestinal-associated mucosal immune response of humans", *J. Gerontol.* **56A**, B218–B223.

Beyer, W., Palache, A. and Baljet, M. (1989) "Antibody induction by influenza vaccines in the elderly: a review of the literature", *Vaccine* **7**, 385–394.

Castle, S.C., Uyemura, K., Crawford, W., Wong, W. and Makinodan, T. (1999) "Antigen presenting cell function is enhanced in healthy elderly", *Mech. Ageing Dev.* **107**, 137–145.

Daniels, C.K., Schmucker, D.L. and Jones, A.L. (1985) "Age-dependent loss of dimeric immunoglobulin A receptors in the liver of the Fischer 344 rat", *J. Immunol.* **134**, 3855–3858.

Daniels, C.L. and Schmucker, D.L. (1987) "Secretory component-dependent binding of immunoglobulin A in the rat, monkey and human: a comparison of intestine and liver", *Hepatology* **7**, 517–521.

Daniels, C.K., Schmucker, D.L., Jones, A.L. and Bazin, H. (1988) "Immunoglobulin A receptor of rat small intestinal enterocytes is unaffected by aging", *Gastroenterology* **94**, 1432–1434.

Daniels, C.K., Perez, P. and Schmucker, D.L. (1993a) "Alterations in CD8 cell distribution in gut-associated lymphoid tissues (GALT) of the aging Fischer 344 rat: SA correlated immunohistochemical and flow cytometric analysis", *Exp. Gerontol.* **28**, 549–555.

Daniels, C.K., Schmucker, D.L. and Irvin, B. (1993b) "Differential IgA responses in the aging Fischer rat following mucosal stimulation with cholera toxin or B subunit", *Aging Immunol. Infect. Dis.* **4**, 95–107.

Ebersole, J., Smith, D. and Taubman, M. (1985) "Secretory immune responses in aging rats. I. Immunoglobulin levels", *Immunology* **56**, 345–350.

Fagiolo, U., Amadori, A., Cozzi, E., Bendo, R., Lama, M., Douglas, A. and Palu, G. (1993) "Humoral and cellular immune response to influenza virus vaccination in aged humans", *Aging* **5**, 451–458.

Farstad, I.N., Halstensen, T.S., Lazarovits, A.I., Norstein, J., Fausa, O. and Brandtzaeg, P. (1995) "Human intestinal B-cell blasts and plasma cells exprss the mucosal homing receptor integrin alpha 4 beta 7", *Scand. J. Immunol.* **42**, 662–672.

Farstad, A.I., Halstensen, T.S., Kvale, D., Fausa, O. and Brandtzaeg, P. (1997) "Topographic distribution of homing receptors on B and T cells in human gut-associated lymphoid tissue", *Am. J. Pathol.* **150**, 187–199.

Freier, S., Eran, M. and Faber, J. (1987) "Effect of cholecystokinin and of its antagonist, of atropine and of food on the release of immunoglobulin A and immunoglobulin G specific antibodies in rat intestine", *Gastroenterology* **93**, 1242–1246.

Fujihashi, K., Koga, T. and McGhee, J. (2000) "Mucosal vaccination and immune responses in the elderly", *Vaccine* **18**, 1675–1680.

Gardby, E. and Lycke, N. (2000) "C-19-deficient mice exhibit poor responsiveness to oral immunization despite evidence of unaltered total IgA levels, germinal centers and IgA isotype switching in Peyer's patches", *Eur. J. Immunol.* **30**, 1861–1871.

Ginaldi, L., Loreto, M.F., Corsi, M.P., Modesti, M. and De Martinis, M. (2001) "Immunosenescence and infectious diseases", *Microbes Infect.* **3**, 851–857.

Gransden, W., Eykyn, S. and Phillips, T. (1994) "Septicaemia in the newborn and elderly", *J. Antimicrob. Chemother.* **34**, 101–119.

Gregoire, C., Zhang, L. and Daniels, C.K. (1992) "Expression of the polymeric immunoglobulin receptor by cultured aged rat hepatocytes", *Gastroenterology* **103**, 296–301.

Jeandel, C., Laurain, M. and Decottignies, F. (1996) "Infectious diarrhea in the aged", *Rev. Prat.* **46**, 184–188.

Kawanishi, H. and Kiely, J. (1987) "Immunoregulatory defects in murine aged Peyer's patches", *Eur. J. Immunol.* **17**, 1223–1228.

Kawanishi, H. and Kiely, J. (1989) "Immune-related alterations in aged gut-associated lymphoid tissue in mice", *Dig. Dis. Sci.* **34**, 175–184.

Lung, T.L., Saurwein-Teissl, M., Parson, W., Schonitzer, G. and Grubeck-Loebenstein, B. (2000) "Unimpaired dendritic cells can be derived from monocytes in old age and can mobilize residual function in senescent T cells", *Vaccine* **18**, 1606–1612.

Medina, S., Del Rio, M., Ferrandez, M., Hernanz, A. and De la Fuente, M. (1998) "Changes with age in the modulation of natural killer activity of murine leukocytes by gastrin-relasing peptide, neuropeptide Y and sulfated cholecystokinin octapeptide", *Neuropeptides* **32**, 549–555.

Medina, S., Del Rio, M., Cuadra, B.D., Guayerbas, N. and De la Fuente, M. (1999) "Age-related changes in the modulatory action of gastrin-releasing peptide, neuropeptide Y and sulfated cholecystokinin octapeptide in the proliferation of murine lymphocytes", *Neuropeptides* **33**, 173–179.

Ogino, T., Miura, S., Komoto, S., Hara, Y., Watanabe, C., Koseki, S., Hokari, R., Nagata, H., Hachimura, S. and Kaminogawa, S. (2001) "Senescence-associated decline of lymphocyte migration in gut-associated lymphoid tissues of rat small intestine", *Gastroenterology* **120**, 3634, (abstract).

Owen, R.L. and Lew, J. (1995) "Gastrointestinal infections in the elderly", In: Surawicz, C. and Owen, R.L., eds, Gastrointestinal and Hepatic Infections (W.B. Saunders, Philadelphia), pp 552–564.

Penn, N., Purkins, L. and Kelleher, J. (1991) "Aging and duodenal mucosal immunity", *Age Ageing* **20**, 33–36.

Powers, D. (1992) "Immunological principles and emerging strategies of vaccination for the elderly", *Am. J. Geriatr. Soc.* **40**, 81–94.

Schmucker, D.L. and Owen, R.L. (1997) "Aging and the gastrointestinal mucosal immune response", *Curr. Opin. Gastroenterol.* **13**, 534–541.

Schmucker, D.L., Gilbert, R., Jones, A.L. and Bazin, H. (1985) "Effect of aging on the hepatobiliary transport of dimeric immunoglobulin A in the male Fischer rat", *Gastroenterology* **88**, 436–443.

Schmucker, D.L., Daniels, C.L., Wang, R.L. and Smith, K. (1988) "Mucosal immune response to cholera toxin in aging rats. I. Antibody and antibody-containing cell response", *Immunology* **64**, 691–695.

Schmucker, D.L., Heyworth, M. and Owen, R.L. (1996) "Impact of aging on gastrointestinal mucosal immunity", *Dig. Dis. Sci.* **41**, 1183–1193.

Schmucker, D.L., Thoreux, K. and Owen, R.L. (2001) "Aging impairs intestinal immunity", *Mech. Ageing Dev.* **122**, 1397–1411.

Takeuchi, M. and Baichwal, V. (1995) "Induction of the gene encoding mucosal vascular addressin cell adhesion molecule 1 by tumor necrosis factor-α is mediated by NF-κB proteins", *Proc. Natl Acad. Sci. USA* **92**, 3561–3565.

Taylor, L., Daniels, C.L. and Schmucker, D.L. (1992) "Aging compromises gastrointestinal mucosal immune response in the rhesus monkey", *Immunology* **75**, 614–618.

Thoreux, K., Owen, R.L. and Schmucker, D.L. (2000) "Intestinal lymphocyte number, migration and antibody secretion in young and old rats", *Immunology* **101**, 161–167.

Vajdy, M. and Lycke, N. (1992) "Cholera toxin adjuvant promotes long-term immunological memory in the gut mucosa to unrelated immunogens after oral immunization", *Immunology* **75**, 488–492.

Van Bezooijen, R., Wang, R.K., Lechner, M.C. and Schmucker, D.L. (1994) "Aging affects the regulation of preferentially expressed liver proteins in male Fischer 344 rats", *Exp. Gerontol.* **29**, 186–196.

Waldmann, R., Bermann, R. and Stone, J. (1987) "Age-dependent antibody response in mice and humans following oral influenza immunization", *J. Clin. Immunol.* **7**, 327–332.

Wick, G., Jansen-Durr, P., Berger, P., Blasko, I. and Grubeck-Loebenstein, B. (2000) "Diseases of aging", *Vaccine* **18**, 1567–1583.

Yoshikawa, T. (2000) "Epidemiology and unique aspects of aging and infectious diseases", *Clin. Infect. Dis.* **30**, 931–933.

